# Delusions of Immortality in a Post-War Society: The Albanian Case

**DOI:** 10.3389/fpsyt.2019.00613

**Published:** 2019-08-23

**Authors:** Gentian Vyshka, Ariel Çomo

**Affiliations:** Faculty of Medicine, University of Medicine in Tirana, Tirana, Albania

**Keywords:** delusions, immortality, WWII, war, psychosis, group dynamics

## Introduction

The Albanian society suffered deep changes and restructuring immediately following WWII. And as if it were not enough, the society was shaken up for decades thereafter. The population was forced to live in a constant collective psychosis of war, with an imaginary ‘enemy’ continuously at the gate, apart from multiple everyday restrictions and an almost complete isolation from the neighboring countries (1, 2).

The Second World War (WWII) in the form it affected Albania, with subsequent invasions of fascist Italians and Nazi Germans, was almost simultaneously tinged with the colors of a civil war. Border skirmishes with Yugoslav partisans took place in the northern areas, sometimes corresponding with the crimes perpetrated from communist Albanians themselves. These factors, along with the Italian–Greek war whose hostilities happened partially on Albanian soil, have obviously rendered the theme of war, martyrs, heroes, and survivors, a highly popular one as part of the public political discourse (2, 3).

The moment when communists started ruling Albania might appear to coincide historically with the end of the Second World War; instead the psychological state of war induced by the regime was obviously not over. The psychosis of the war kept on looming with ebbs and tides for more than forty years (1945–1990), and has almost always been present in the psychological and physical environment, and unrelentingly broadcasted by official mass media (4).

The psychiatric and mental consequences in individuals following war, post-war, chronic exposure to hostilities, ill-treatment, and torture related to belligerencies, have been studied among Holocaust survivors (5). Other authors have enlarged their scope of study, and even of the diagnostic notions depicting war-related disorders (6, 7). However, large-scale studies on war impact are usually limited to a simple PTSD model of impact, without consideration of the complex impact of an imaginary of impending war, leading to continuous fear, avoidance behavior, and maladaptive coping styles that will potentially affect generations of a post-war society, even when there is overlapping symptomatology between PTSD and this complex changes in the large group's members (8).

## The Post War as a Continuity of Everlasting Hostilities

The communist propaganda took particular care to put pressure on the general population by, as noted before, continuously evoking the fear of an impending aggression. The case of Albania might be not unique; however, different strategies of influencing general opinion and fomenting feelings of incessant aggression have been documented during the cold war in the region (9).

One of the most productive and efficient strategies to make Albanians believe that the war was “not over,” or at least imminent, were the graphic representations recalling the war. This mission was performed through flooding the country with tombstones and headstones of the dead patriots, mainly from the Second World War. The extension of these graphic monsters inside the territory was immense, and the writings on these stony premises evoked the incessant presence of the heroes: these are and must be immortal, i.e. still alive and among the people.

The number of stone monuments (‘*lapidar*’ – from Latin *lapis*, lapid – ’stone’) exceeded 500 all over the Albanian soil, and was clearly decreasing after 1990 due to degradation, removal or deliberate destruction (10). Nevertheless, this massive presence for almost forty years (their construction started almost immediately after the end of WWII) might have played an important role into shaping the general opinion approach toward the alleged immortality of heroes.

The stone monuments were obviously not the only way that the official propaganda kept on imposing beliefs on the population (**Figures 1** and **2**, **Supplementary material**). The idea of heroes’ immortality while fighting against the enemy, limited in reality mostly to the WWII years, has been a preferred subject for the production of movies, media, and literature (11). Working always under a very strict censorship, almost the entirety of all authors’ works, papers, movies, and pictures, highlighted mainly the official policy of the governing party. Some free space (if any) could have been found solely in the interactive discourse between the official doctrine and the individual contributions to the public discourse (12).

The indoctrination and the perseverance on the theme of war, mixed up with a sense of glorification towards the dead, spread all over the society. In an attempt to incessantly control the public opinion, war (mainly WWII) and its aftermath were immortalized in sculptures, pictures, movies, and written media of all forms; from everyday journalism kitsch to serious literary works of hundreds of pages.

As the country entered an unprecedented period of state atheism (1967–1991), religious views were silenced or merely transformed, in a strongly death-denying culture (13). Some behavioral models and changes had therefore time to bloom into a delusional state and collective state of mind we perceive as a form of collective “war psychosis”. The alarmism spread from continuous imaginary threats that the communist regime reported on a daily basis, the militarization of the society with a huge amount of micro bunkers covering Albania and built within a psychotic dictator’s regime, the morning sirens that announced foreign assaults that never took place; all these and other factors strongly influenced post-war generations (**Figure 3** and **Supplementary material**). Behavioral models and life experiences were therefore influenced and distorted — not merely as a cultural trope.

## An Immortal, Albeit Hostile General

The novel “*The General of the Dead Army*” of Ismail Kadare that we want to take as an example for the symbols and delusions of immortality was published in Albanian in 1963, and thereafter translated in several languages and re-published, with a first translation in French some seven years after the launch. The plot is focused on the everyday miseries and passions of an Italian general, visiting Albania with the task of exhuming and repatriating the remains of the soldiers, fallen during the lost battles of his country’s army that invaded Albanian soil during WWII. The novel has been adapted as a movie in three versions, two different Albanian productions and one French–Italian coproduction (14), so it can be used for analysis of the public discourse.

A particular, obviously pathological state of mind is depicted, especially through identification and misidentification based delusions that gradually occupy the psychic horizon of the main actor, the *General*. Believing himself to be immortal and identifying his own image with that of a lost corpse, specifically that of the colonel Z, the *General* ambivalently pursues an insistent search of the fate of the dead colonel, and still conceals the remains of the latter, through dispersing the bones immediately after uncovering his skeleton. This psychic drama of the delusional belief in immortality, the particular setting of a post-war environment, and the figure of an army general collecting exhumed skeletons, are the main components of this complex history of psychotic despair.

The history of exhuming soldiers’ corpses from Albanian soil has been a long one, and recently flared up again with the re-burial of Greek soldiers fallen during the Italian–Greek war of ‘40ies. Italian, British, and German soldiers have had also their historical share and war cemeteries have been built up to honor respective sacrifices (**Figure 4** and **Supplementary material**).

The novel *The General of the Dead Army* was an artistic mirror not only of the repatriation of Italian soldiers’ remains. This last operation was organized and performed some ten years after the WWII was over. The person in charge of the real mission was a military chaplain in the ecclesiastic hierarchy; whereas the writer in the novel separates the man in charge into two different characters: that of the *General* and that of the *Priest*.

Uncovering skeletons and packing human remains is obviously not a simple and unemotional job. The *General* itself will endure the hardship of digging into the past. The hostilities might have been in reality been over, but the atmosphere encircling post-war life was not pacific. In a desperate attempt, focused to uncover the remains of *colonel Z*, the *General* identifies himself with the lost colonel. He starts flirting with his beautiful widow, and through undergoing a delusive process of identification with the lost corpse of the colonel, the acting character (*the General*) goes through nightmares, flashbacks, insomnia and delusional fantasies about a war he never experienced directly. After the serendipitous discovery of the remains of *colonel Z*, he decides to throw out the sack with the bones: at that point, the delusion was terminated. Unable to accept the incorrect and delusional identification, *the General* could not stand the idea, that after all, he was only identifying himself with a person killed during the WWII at least a decade before. The belief in immortality persisted for at least two hundred twenty pages of the novel of about two hundred sixty; the lines below describe the moments when the *General* gets rid of the remains of a corpse with whom he identified himself for a long time, till the uncovering of the bones corroded the delusion:

*“…The general stumbled form the second time over the sack.*

It’s this sack, he thought suddenly. It’s this sack that’s the trouble. It’s almost done for us once tonight. Up until now everything had been going perfectly, but now this sinister sack has forced its way into our lives and everything is going wrong!

“It’s this sack that’s put a jinx on us,” he said out loud.

“What did you say?” the priest answered.

“I say that this sack is bringing us bad luck,” the general repeated.

And as he spoke he gave it a vindictive push with his foot. The sack tumbled down the slope and fell with a resounding thwack into the water flowing at the bottom…” (15).

## The Rise and the Fall of a Delusion

It might seem that the novel *The General of the Dead Army* is a solitary reflection of the post WWII Albanian society vis-à-vis the recent past. Instead, partly reckoning with the legacy of Italy’s fascism and the ravages of the Italian invasion and occupation, the writer deals a lot – although implicitly – with the Albanian present (16). Writing under socialism was not an easy job; however, the regime itself used and widely abused the potential of arts and literature to act as mass media, and influence the general opinion, modulate beliefs, and shape emotional group processes (12, 17).

The literature and arts in the post WWII Albania were state-sponsored and therefore, deeply identified with the official ideology. The novel ‘*The General of the Dead Army’* (‘The General’) was one of the most successful and widely read; it was included in the study of literature at almost all curricula covering levels from the obligatory education to the Universities. All together, the bulk of written literature and graphic arts produced and sponsored, reflecting the war heroism and its aftermath, aimed as noted before at creating the subconscious feeling of immortal conquerors.

In order to complete the illusive immortality paradigm, the presence of the enemy needed to be kept uninterrupted, and obviously threatening. The *General* dealt with exhuming corpses of his nation’s army that was defeated in Albanian soil during WWII, but through a continuous emotional process, the *General* itself underwent a delusional experience. While flirting with the widow of the dead *Colonel Z.* and vesting himself as his reincarnation, he was dealing right from the start with a hostile, if not very unusual environment described with terms close to psychotic imagery: **Table 1** reproduces some of the terms depicting the perceived environment.

**Table 1:** Quotes from the text (15).

Page	
***Describing a threatening, hostile environment***	
3	Foreign soil
3	*Menacing* mountains
9	From graveyard to graveyard
9	His campaign against the mud
42	…slits were vertical then the little forts had a *cruel*, menacing expression
67	The circle of bystanders was gazing at them with *popping* eyes
203	Generals always inspire respect
***The General and his counterpart: emotional experience and physical representation***	
4	Feeling of pride
5	…face devoid of all expression
6	…companion’s *taciturnity*
11	*Expressionless* features
11	Absent gaze
12	…felt alarm run through every fibre of his being
19	…severe profile and impassive, *masklike* features
43	*Indecipherable* expression in the villagers’ eyes
43	We remind them of the invasion…
53	Heavens! Anyone would think I’m having *hallucinations*…
125	“A *nightmare*,” the priest said. “Another night I saw Colonel Z. in a dream…”
137	…even a sort of *evil spell*, something sinister anyway, dogging this work of ours…
138	Sense of *oppression*
217	I must sleep, he thought. *Sleep, sleep …* I must sleep at all costs
***Words from the afterworld***	
14	Don’t worry, your grave’s going to be deep, really deep
14	…not to leave any clues behind, for fear someone might just notice something and *dig his body up* again.
61	…I have crossed over into a kingdom of bones, of pure calcium
77	…you’ll die like a dog and no one will be able to recognize that *carcass of yours*
83	Such *exaggerated attachment* to a husband who’s been dead twenty years…
88	…any greater satisfaction for an old soldier than that of pulling his old enemies back up out their graves? It’s like *a sort of extension of the war*

The quotes and pages of the **Table 1** are selected from the English Version translated by Derek Coltman (Vintage Books, 2008) (15).

The menacing mountains and the foreign soil were the background to a hard physical task (page 3) because the *General* had the task of running from a graveyard to the other, in his desperate campaign against the mud threatening the excavations (page 9) (15). His counterpart, the *Priest* who is the companion on this grizzly pilgrimage, will mirror some ambiguous feeling that probably are actually those of the main character – the *General* itself.

Some emotional reactions in this process are indirectly described through the gaze and the facial features of workers employed to perform exhuming work, or bystanders that curiously follow up the *General* during his journey: **Table 1**.

The feeling of *pride of the General* (page 4) will be soon overshadowed by the *taciturnity* of the *Priest*, his companion (page 6); but this is just the beginning of the flourishing delusional situation. The *General* himself speaks about hallucinations on page 53; some seventy pages further he has a *nightmare* while dreaming of the dead (yet immortal) *Colonel* *Z* (page 125). Some kind of *‘evil spell’* seems to control the psyche of the main character, as the sense of *oppression* (page 138) is thereafter replaced by a severe loss of sleep (page 217).

Not less interesting are the dilemmas and thoughts coming from the afterworld ghosts of soldiers, inserted in italics inside the chapters of the novel, sometimes interrupting the main flow of the storytelling (**Table 1**).

The author himself will at the end present the idea of the everlasting war: the exhumation – if not profanation of graves – can be seen as *a form of extension of the war* (page 88).

All these written materials; and other art works reproducing (even fictionally) the WWII and its aftermath, have as noted before been omnipresent part of the Albanian public discourse: hence the psychological potential of keeping the hostilities alive even when there was no war to fight (18). The enemy was there: exhumed and therefore still able to fight and cause damage. The immortality of heroes (partisans) could not stand firm if this page of history was closed. The never-ending war has deeply influenced society and its generations. Several times, authors have suggested that the future of Albania even after the death of the communist dictator would have been conditioned more from its past, rather than from present international developments (19).

## Conclusive Remarks

According to DSM-5 delusions are fixed beliefs, not amenable to change in light of conflicting evidence (20). Abundant psychological and psychiatric research has considered post-war settings, with post-traumatic stress disorder being probably the main theme. However, when reporting on “war neurosis” (in the older psychiatric terminology), some authors, referring particularly to Balkan culture and setting, have even talked about a specific ‘partisan hysteria’ (6, 7, 21).

Cotard’s syndrome is a rare and uncommon syndrome, which is characterized by various symptoms such as nihilistic delusions, hypochondriacal delusions, delusions of immortality, depression, and anxiety (22). Other forms such as delusions of missing organs, “walking corpse” syndrome, denial of existence and ideas of damnation might be part of the same spectrum, with some authors blaming diverse neurological conditions for this unusual psychopathology (23). From the very wide notion of the delusional disorders, Cotard syndrome is considered as ‘monothematic’ with contributing factors that influence two levels: the experiential and the inferential (24). Very close to each other, there is an interesting practical explanatory scheme offered by Ramachandran and Blakeslee (24) for Cotard and Capgras delusions.

Its coexistence with other forms of delusions might be quite well possible within the background of a florid psychopathology (25). The fictional case of a psychotic *General* dealing with the hard work of exhuming and repatriating soldiers’ remains from a recently fought war is just an illustration of how the propaganda fomented delusions.

Assmann discusses the concept of *pietas* referring to religious rituals in the Ancient Greek literature, and the concept of *fama* defined as the glorious memento of individuals after death and the immortalizing of the name of deceased (26). Greek epos is a living sign of how heroes are considered immortal, be that in the pre- or post-Homeric; with the cult of heroes if not being the same, coming very close to the cult of gods (27).

The Balkan area and other countries that once were part of the Ottoman empire have been of great interest to psychology and psychiatry. In fact, continuous civil and inter-ethnic wars culminating sometimes to genocide have impressively influenced the collective consciousness. Vamık Volkan has been one of the most prolific authors on this issue. He suggested the term of ‘chosen trauma’, namely the shared mental representation of a massive trauma that the group’s ancestors reportedly suffered at the hand of an enemy kept artificially alive (28). While commenting on monuments in Eastern Europe, he has underscored their function to reawaken chosen traumata through group symbols, manipulate groups, and induce group regression as a quasi-psychotic state to justify future aggression and wars (28, 29).

The communist regime in Albania suppressed religion but the virtual immortality it granted to the war heroes probably contributed in turn to a new form of religion. Other authors have also commented on the fact that humans intuitively believe that they survive death, although from a quite different perspective (30). A thorough scrutiny of death rituals in the Balkan area might offer further evidence of distorted perceptions vis-à-vis deceased persons and death as a process. A good example are the death rituals in the Greek Mani region described by Fermor where bones are exhumed and heroic poems are recited to keep the memory of dead warriors (and sometimes other persons) alive (31, 32).

In one of the most popular recent (1995) movies, the Bosnian born Serbian film-maker Emir Kusturica described a group of former partisan war heroes and their supporters, living in a psychotic underground post-communist micro society, producing military weapons, while the leaders convinced them that the war was actually not over in order to himself remain in power (33).

In summary, post-WWII Albanian society has been living under the burden of an unrelenting war psychosis. The enemy was “at the gates”, and if not ready to assault, this assault would be seen as just a question of time. The huge number of concrete bunkers (more than one hundred seventy thousands) scattered over the small territory of the Mediterranean country during the time of the communist regime reflecting Hoxha’s paranoiac position when leader of the country, contributed to the creation of a state of siege. Literature, graphic arts, cinematography and all other mass media were serving the same idea that the war was not over. Hence the heroes were still alive: their immortality meant that the enemy was still a continuous threat, with the society called to identify with the heroes in the defense of the country.

## Author Contributions

All the authors contributed to the conceptualization and the drafting of the paper and they critically reviewed the manuscript.

## Conflict of Interest Statement

The authors declare that the research was conducted in the absence of any commercial or financial relationships that could be construed as a potential conflict of interest.
