# Supplementary material for: Delusions of Immortality in a Post-War Society: The Albanian Case
Source: Front Psychiatry. 2019 Aug 23;10:613. doi: 10.3389/fpsyt.2019.00613 (PMC6716475; doi:10.3389/fpsyt.2019.00613)
Supplement: Supplementary file 1 [file DataSheet_1.doc]

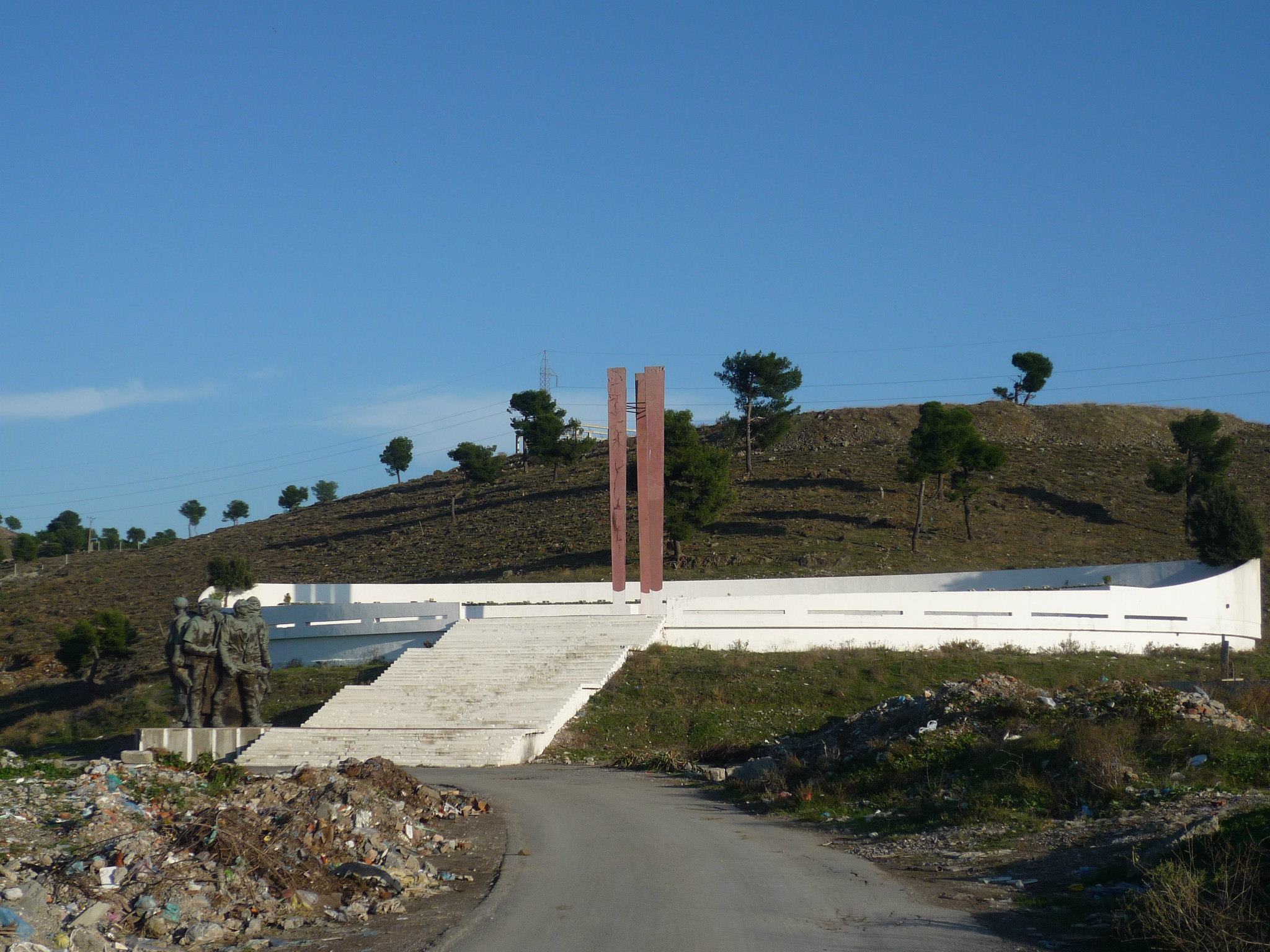


***Figure 1****: One of the countless stone monuments that spread over the Albanian territory in the memory of WWII heroes. Some stone monuments representing partisans at the left side of the image actually also reflect the erosion that the time has afflicted to the structure and its importance: heroes stand up close to a garbage pile* (courtesy of *Albanian Film Archive,* Tirana).


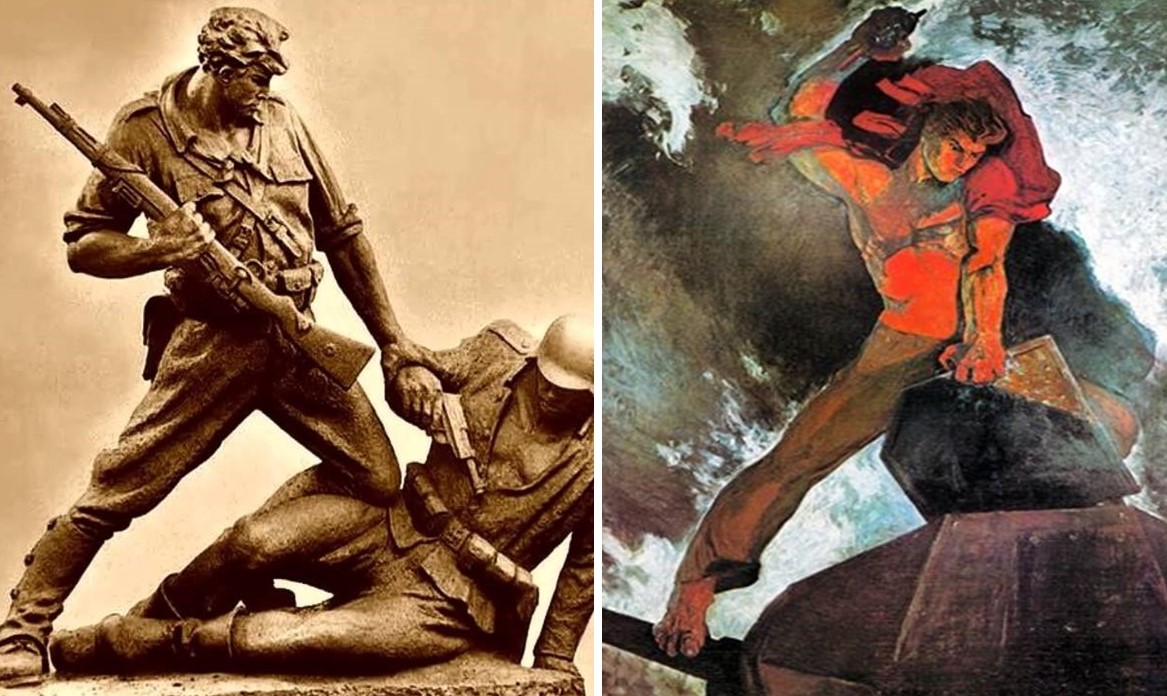


***Figure 2:*** *Graphic art from Albanian authors covering the war theme;* ***left inset:*** *a sculpture with a partisan defeating a Nazi soldier (artwork of Odhise Paskali, a renown Albanian sculptor);* ***right inset:*** *a picture reproducing a guerilla act of bombing an enemy tank (artwork of Sali Shijaku, a renown Albanian painter). The enemy was always there; defeated, but still alive and capable of doing evil things* (courtesy of *Albanian Film Archive*, Tirana).


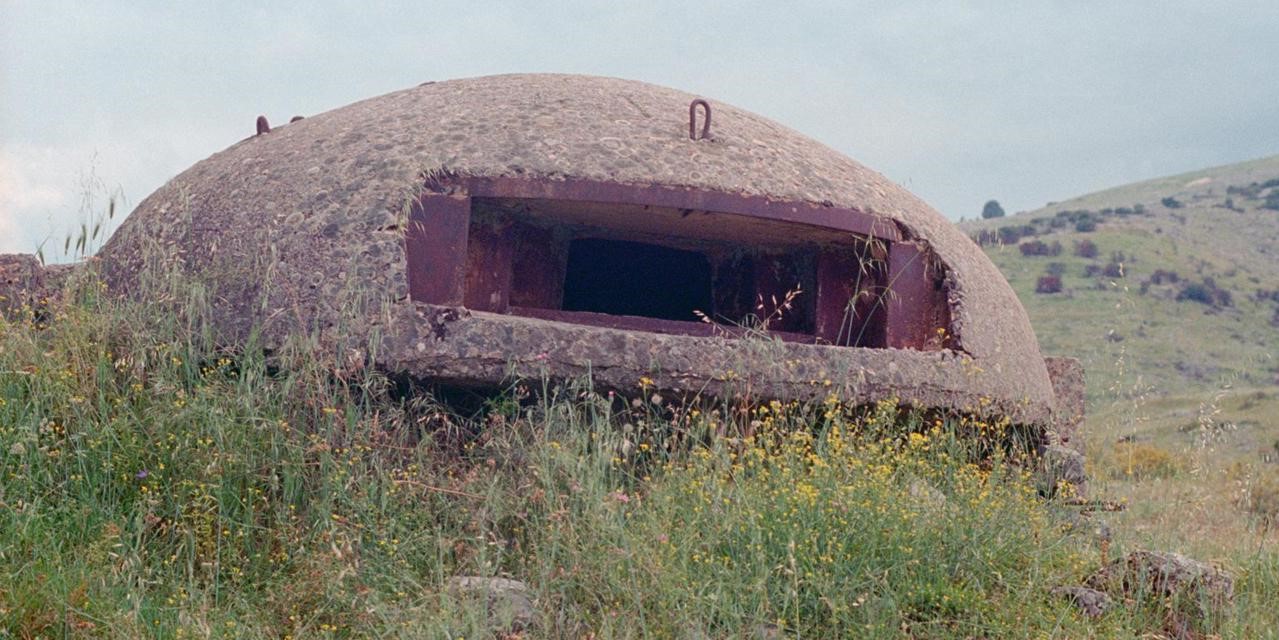


***Figure 3:*** *Unofficial data suggest that approximately one hundred and seventy thousand bunkers were distributed all over the Albanian soil. Their density was higher in the border areas and close to the seashore. This huge amount of concrete blocks never served for military purposes, and irreversibly polluted the landscape* (*photo:* Gentian Vyshka).


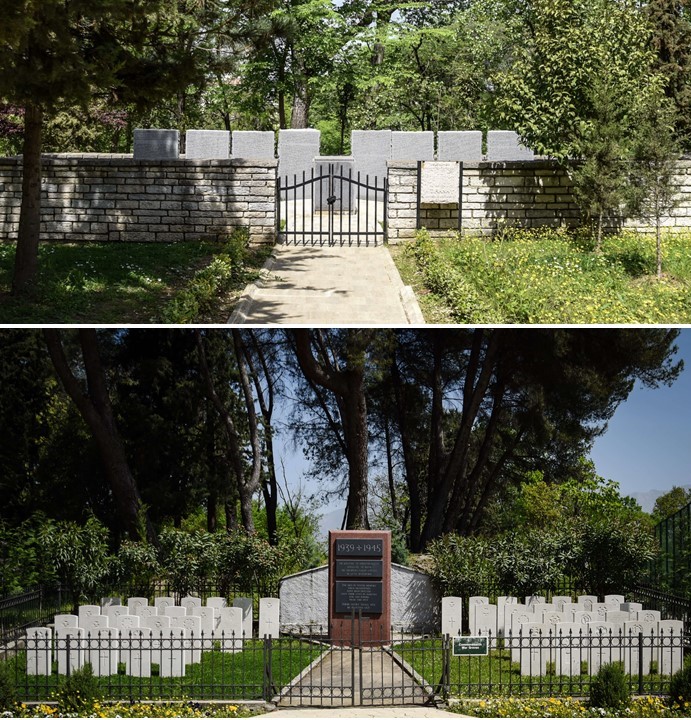


***Figure 4:*** *Cemeteries of German (****above inset****) and British soldiers* ***(below inset)*** *in the hilly suburb of Tirana, Albania* (*photos:* Gentian Vyshka).
